# A developing world experience with distal foot amputations for diabetic limb salvage

**DOI:** 10.3402/dfa.v4i0.22477

**Published:** 2013-10-21

**Authors:** Omer Salahuddin, Muhammad Azhar, Aqsa Imtiaz, Munawer Latif

**Affiliations:** 1 Department of Plastic Surgery, Shifa International Hospital, Islamabad, Pakistan; 2 Department of Surgery, Pakistan Ordnance Factories Hospital, Wah Cantt, Pakistan

**Keywords:** diabetic foot, limb salvage, glycemic control, amputations, infections

## Abstract

**Objectives:**

To evaluate the functional outcome, morbidity, and viability of foot salvage in diabetic patients.

**Materials and methods:**

This prospective case series was conducted from March 2007 to December 2012 at the department of surgery Pakistan Ordnance Factories Hospital, Wah Cantt, Pakistan. 123 males and 26 female patients were included in the study. All the patients were treated after getting admitted in the hospital and wounds were managed with daily dressings, nursing care and debridement of necrotic tissue with adequate antibiotic coverage.

**Results:**

In total, 149 patients (mean age: 56±7.52 years) with 171 amputations were included in the study. The mean duration of diabetes mellitus (DM) was 9±4.43 years. Ninety-seven percent of the patients were diagnosed with type 2 DM. Wound debridement was performed under general anesthesia in 48 (33.2%) patients, whereas local anesthesia was used for the rest of the patients after having good glycemic control and improvement in general health. The most common pathogen isolated from the infected wounds was *Staphylococcus aureus* in approximately 46% cases. Regarding the types of amputation, partial toe amputation was performed in 21 (12.2%) cases, second-toe amputation in 60 (35%) cases, hallux amputation in 41 (24%) cases, multiple toe amputations in 29 (17%) cases, bilateral feet involvement was observed in 16 (9.3%) cases, and transmetatarsal amputation was performed in 4 (2.3%) cases. The wounds healed well except in 19 cases where amputation had to be revised to a more proximal level. Thirty-nine patients died during the study period: 3 died of wound-related complications and 36 died of systemic complications.

**Conclusion:**

With the ever-increasing epidemic of DM, the number of patients with diabetic foot ulcers has also significantly risen. Early surgical management with good glycemic control and foot care with close monitoring can decrease amputations and thus foot salvage can be successfully achieved.

Diabetic foot ulcers can occur because of a combination of neuropathy, microvascular angiopathy, mechanical stress, and uncontrolled blood glucose levels (1). Diabetic foot ulcerations are one of the leading causes of morbidity leading to infection and ultimately amputation. Infection can increase the amputation rate to almost double the number in non-infected ulcers and more than 50% of non-traumatic amputations occur because of diabetes mellitus (DM) (2). Delayed diagnosis and extensive soft-tissue compromise leads to poor prognosis (3). Diabetic neuropathy with concomitant soft-tissue loss and increased susceptibility to infection have been considered a major cause for the development of diabetic foot gangrene (4).

Absent pulses, plantar foot abscesses, infection, cellulitis above the ankle, and localized gangrene do not necessarily require entire foot amputation if the foot is otherwise viable. These cases can be treated by repeated operative debridement and resection of distal osseous and soft-tissue structures, daily dressings, strict glycemic control and intravenous antibiotic therapy for eradication of infection. Whenever a viable bleeding tissue is present, the surgeon should judge the distal amputation level and consider foot salvage. One can retain the potential for weight bearing if the limb can be salvaged by simply performing the limited distal amputation. The limited amputation for limb salvage includes resection of one or more toes, ray (metatarsal and toe) amputation, and transmetatarsal amputation.

Pakistan has a high prevalence of diabetes (5–7), and amputation rates are high because of late referrals, poor medical facilities, and decreased awareness regarding foot care (8). Major limb amputations can be prevented by early referral, prompt management and foot-care awareness because diabetic neuropathy and infection is the leading cause of gangrene in the developing world as compared to vasculopathy which is a major issue in the western world (9). The main objective of this study was to highlight the importance of early and aggressive management and viability of limb after distal or forefoot amputation.

## Materials and methods

This prospective case series was conducted at Surgical Unit I, Pakistan Ordnance Factories Hospital Wah Cantt, Pakistan, in close collaboration with the diabetic clinic for strict glycemic control from March 2007 to December 2012. After approval from a local ethical committee, all patients from the outpatient or emergency department requiring admission and surgical debridement with distal or forefoot amputation were included in the study. Patients with a potential for amputation at or above the level of calcaneus on their first admission were excluded from the study.

A thorough history was taken regarding the duration of diabetes, insulin dependence, duration of symptoms and other diabetes related systemic complications. Detailed examination of the foot was performed for areas and types of ulceration, infection, purulent discharge, gangrene and atrophic changes. Circulation was checked by palpating the dorsalis pedis and posterior tibial arteries. Doppler was used in patients where pulses were not palpable. Ankle brachial pressure index was measured and those having <0.8 were excluded from the study. Plain foot radiographs were performed in all cases of infected ulcers to rule out osteomyelitis. Complete blood count, urine analysis, renal profile and electrocardiograms were ordered. A Diabetologist was also involved in all the cases for the control of diabetes. Surgical treatment was performed by debridement of all non-viable tissue, daily dressings and close examination of the wound. Wound debridement was performed under general anesthesia in 48 (33.2%) patients whereas local anesthesia was used for the rest of the patients. Tissue cultures were sent for aerobic and anaerobic organisms and broad-spectrum antibiotics were started at the time of admission. Surgical treatment including debridement and minor amputations were noted. Patients were then followed for at least 6 months for viability of limb salvage and reamputation.

## Results

**Fig. 1:**
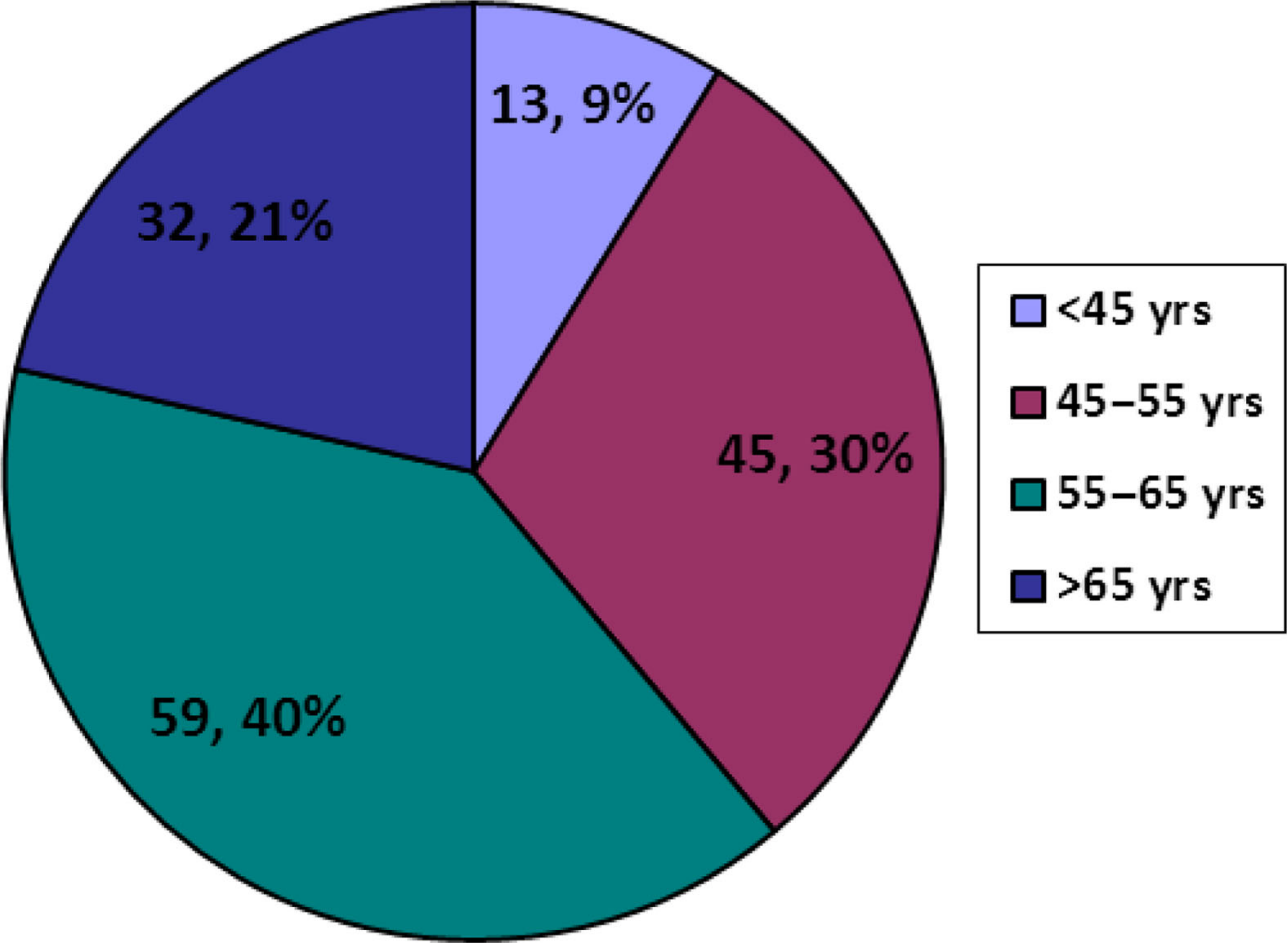
Age distribution of patients.

In total, 149 patients with 171 amputations were included in the study. There were 123 males and 26 females with male to female ratio of 4.7:1. The age range was 41–76 years with a mean age of 56±7.52 years (Fig. 1). All patients were established diabetics with few known diabetics for the last 15 years. The mean duration of DM was 9±4.43 years. Ninety-seven patients were diagnosed as type 2 diabetics. Blood sugar levels were well controlled in all the cases by either oral hypoglycemic agents or injection of insulin prescribed by Diabetologist.

The most common pathogen isolated was *Staphylococcus aureus* in approximately 46% cases. Other organisms isolated were *Streptococcus* (4%), *Escherichia coli* (15%) and *Pseudomonas aeruginosa* (13%). Thirty patients had polymicrobial cultures, whereas11 patients had no growth after 48 hours of incubation. Infection was treated early and vigorously with broad-spectrum antibiotics initially and followed by culture-specific antibiotic coverage.

**Fig. 2:**
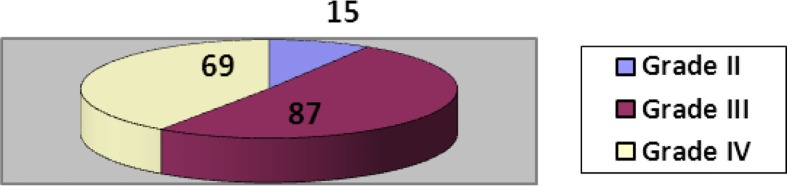
Frequency of Wagner grades of diabetic foot ulcers.

**Fig. 3:**
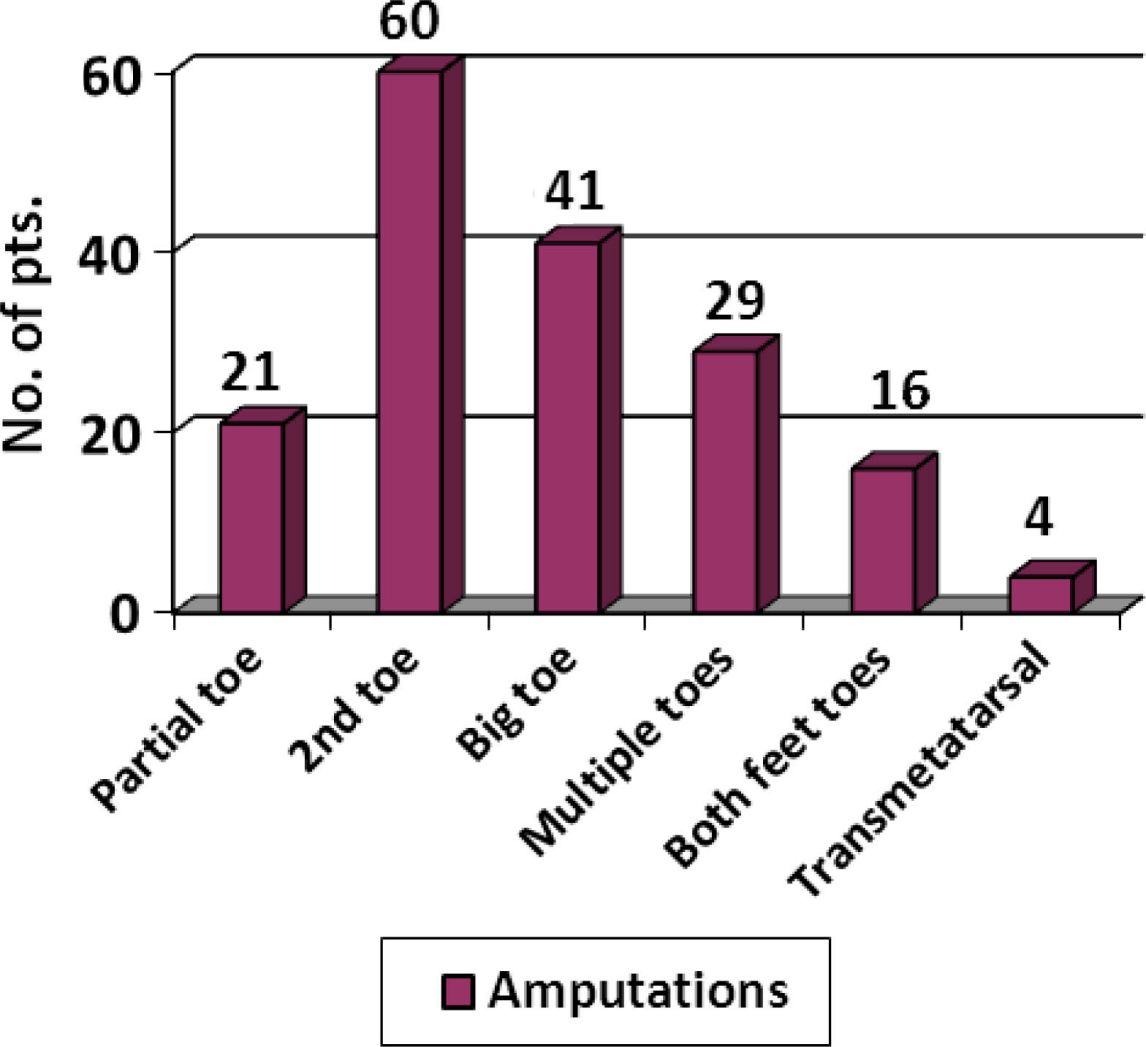
Types of diabetic foot amputations.

**Fig. 4:**
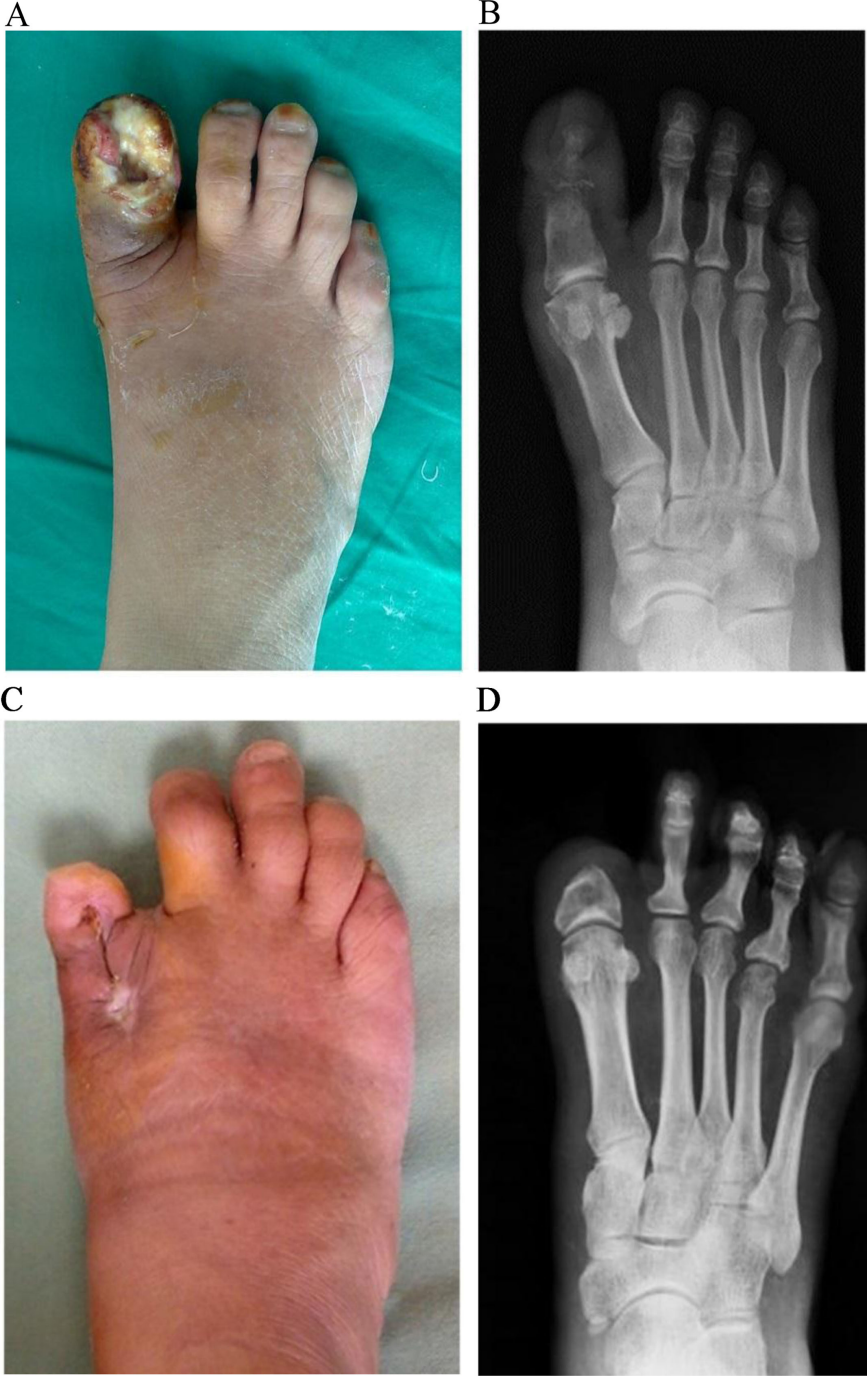
Pre-operative clinical (A) and radiographic (B) views of a big toe (hallux) gangrene in a 58-year-old type 2 diabetic female, with a healed wound 6 months after surgical debridement and partial hallux amputation (C, D).

**Fig. 5:**
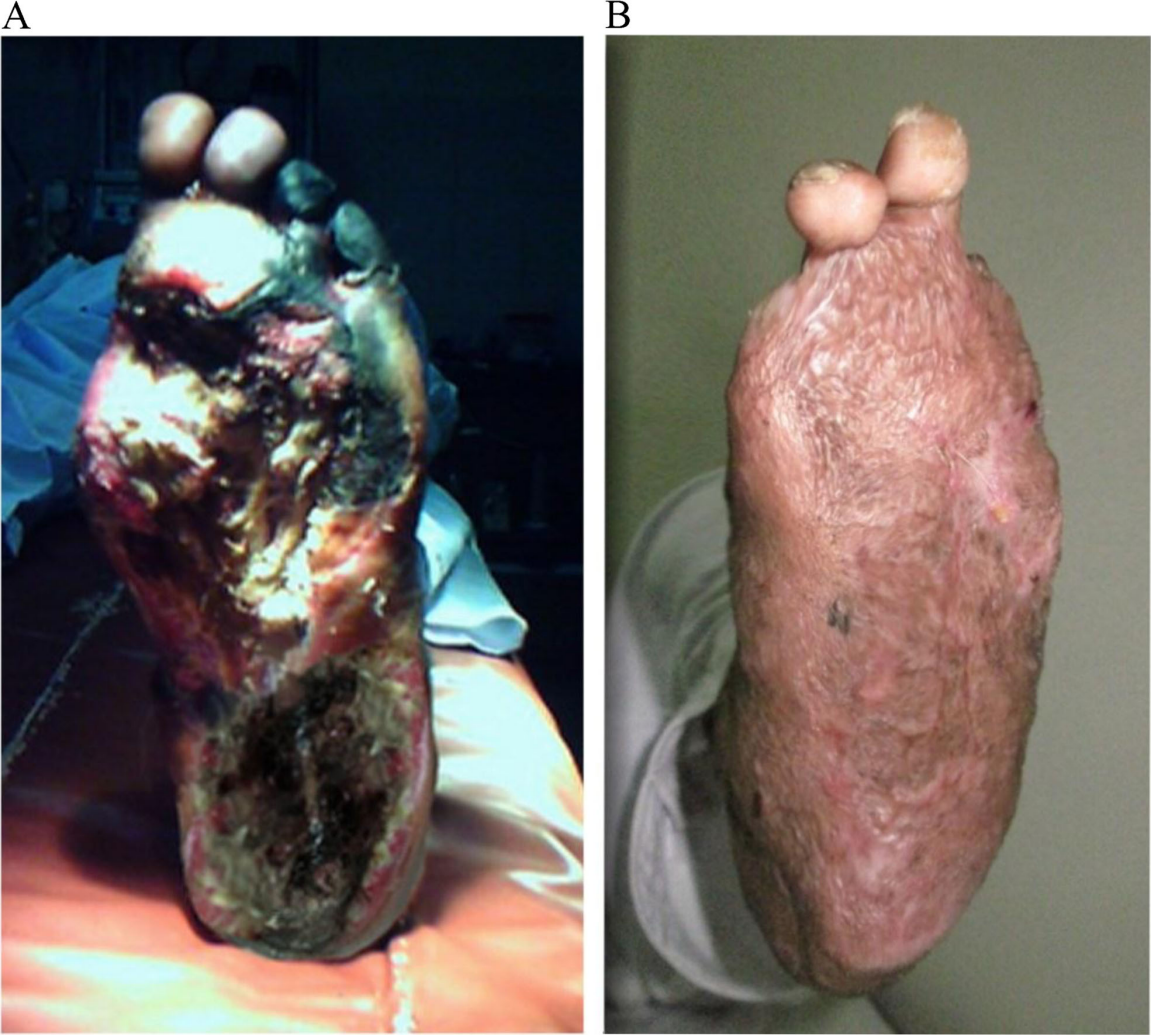
Extensive necrosis and toe gangrene in a 49-year-old male (A), with a healed wound after surgical debridement, negative-pressure wound therapy, and split thickness skin graft at 5 months post-operatively (B).

Diabetic ulcers were classified according to Wagner's classification and their frequency is shown in Fig. 2. The types of amputations performed are shown in Fig. 3. Partial toe amputations (preservation of base of the proximal phalanx) were performed in patients having ulcers at the first and second-toe distal tips (Fig. 4). Ray amputations were performed in all cases where infection involved the base of the toe. Ninety-eight percent of the wounds were closed primarily or by secondary intention while the rest of the wounds were covered with a split thickness skin graft (Fig. 5). The wounds averaged 6 weeks to heal completely. Patients were followed in the outpatient department weekly for the first month, fortnightly for the next 3 months and then monthly for at least 6 months. The wounds healed well except in 19 cases. Five patients had a Chopart's amputation, 2 underwent a Syme's amputation, 11 underwent a below knee amputation and 1 had an above knee amputation. New ulcers were treated aggressively with local wound-care dressings, good glycemic control and minor debridements. Thirty-nine patients died during the study period: 3 died of wound-related complications and 36 died of systemic complications.

## Discussion

Pakistan ranks seventh among countries with highly prevalent diabetes and DM has become an epidemic (10). High prevalence of diabetes, glucose intolerance and diabetes-related complications in the developing countries like Pakistan have emerged as a major social and economic burden. Diabetic patients have a 15-fold higher risk of amputation but half of these amputations can be prevented if these patients can be treated early, educated about foot care and have good glycemic control (11).

The major cause of amputation in DM is the development of a foot ulcer. The pathogenesis of a foot ulcer in the diabetic foot can be because of lack of sensation, poor perfusion and infection. Neuropathy develops slowly over the years and once the foot ulcer develops, infection can worsen the condition. Vascular compromise can further deteriorate the clinical case scenario and worsen the potential of healing. Management should focus on strict glycemic control, meticulous debridement, adequate antibiotic coverage, foot-care awareness and preservation of all viable tissues.

Diabetic foot ulcerations usually occur during the fifth to seventh decade of life (12). In our study, 61% patients were more than 55 years similar to other studies in Pakistan (13, 14). Hamalainen et al. (15) mentioned a higher rate of amputation among males but few studies did not label it as a significant finding (16). In our study, the male-to-female ratio was 4.7:1, highlighting the male predominance. The severity or grade of diabetic foot ulcers is one of the most important factors to determine the treatment plan (17). Many classification systems have been proposed such as the depth – ischemia classification and size, area, depth classification to assess and classify the severity of diabetic ulcers. Among all classification systems, Wagner's classification is the most practical and simple with a prediction of healing and possibility of amputation. More than 90% of the cases were Wagner grade III and IV. This correlates with studies by Aziz et al. (18) and Munnawar et al. (14) Infection must be controlled with antibiotic coverage and broad-spectrum antibiotics can be started as soon as signs and symptoms of infection appear. Culture-specific antibiotics can then replace the empirical therapy for better results. In our study, the most common organism isolated was *Staphylococcus aureus*. This correlates with other local (14, 18) and international studies (16, 19).

Surgical management of diabetic foot infections includes early and aggressive debridement of all potentially non-viable tissue. Multiple debridements are usually required and careful monitoring of patients along with foot-care programs is the key to successful limb salvage. Foot infection is one of the major causes of major limb amputation. Infection can spread from the distal foot proximally to the leg through the tarsal tunnel and tendon sheaths. Adequate surgical debridement and multiple local wound-care dressings can prevent major limb amputation. Early surgery and foot-care programs have decreased major amputations up to 80% in developed countries (20). In our study, subsequent amputation rate was 11% which correlates with studies by Frykberg et al. (21), Al-Wahbi (22), and Cavanagh et al. (23).

Distal foot amputations if required must be planned to provide adequate treatment and minimize the requirement of future amputation. Amputation levels can be determined by the extent of ulceration, infection and blood supply of the toe. Partial toe amputation (preservation of base of the proximal phalanx) is sometimes preferable for patients undergoing elective amputation. In hallux amputations, preservation of the big toe is beneficial for terminal gait phase as it maintains attachment of the flexor hallucis brevis, whereas hallux valgus is prevented by the retained segment of the proximal phalanx in 2nd toe amputations (24). It can also be beneficial for patients with hammer toe deformities. Ray (metatarsal and toe) amputation is a better option than metatarsal disarticulation when the necrosis has extended to the base of the proximal phalanx of the toe. In our study 21 patients underwent partial toe amputation while ray amputation was performed in 130 patients.

The multidisciplinary approach to limb salvage cannot be overemphasized. Obviously, strict blood glucose control during the course of antibiotic therapy for infection should involve the physician's opinion. As distal foot amputation is almost always considered for patients who have complex medical and surgical conditions, several specialists both surgical and non-surgical must be called upon to optimize care in the limb salvage effort.

## Conclusion

Preservation of the limb function without endangering the patient must be the goal of treating ischemic and infectious diabetic conditions of the lower extremity. A safe approach can incorporate the concepts that infection should be treated with surgical debridement and proper antibiotic coverage. Foot-care programs must be launched in the developing countries to counteract the epidemic of DM and its related complications. Patient education, early referral and surgical expertise can lead to functional limb salvage with acceptable outcome.
